# The First Experience of *Ex-Vivo* Lung Perfusion (EVLP) in Iran: An Effective Method to Increase Suitable Lung for Transplantation

**Published:** 2016-11-01

**Authors:** S. Shafaghi, K. Najafizadeh, K. Sheikhy, Z. Ansari Aval, B. Farzanegan, Y. Mafhoomi, Z. Faghih Abdollahi, H. Emami, E. Mortaz, M. Porabdollah, A. Jahangiri Fard, M. Nikobayan Safaei, A. Slama, C. Aigner, F. S. Hosseini-Baharanchi, A. Abbasi Dezfuli

**Affiliations:** 1 *Lung Transplant Research Center, NRITLD, Shahid Beheshti University of Medical Sciences, Tehran, Iran*; 2 *Organ Transplantation Office, Ministry of Health and Medical Education, Iran*; 3 *Tracheal Diseases Research Center, NRITLD, Shahid Beheshti University of Medical Sciences, Tehran, Iran*; 4 *Tobacco Prevention and Control Research Center, NRITLD, Shahid Beheshti University of Medical Sciences, Tehran, Iran*; 5 *Cell and Molecular Biology Group, Airways Disease Section, National Heart and Lung Institute, Faculty of Medicine, Imperial College London, Dovehouse Street, London, United Kingdom*; 6 *Chronic Respiratory Diseases Research Center, NRITLD, Shahid Beheshti University of Medical Sciences, Tehran, Iran*; 7 *Department of Thoracic Surgery, Medical University of Vienna, Vienna, Austria*; 8 *Minimally Invasive Surgery Research Center (MISRC), Iran University of Medical Sciences, Tehran, Iran*

**Keywords:** Lung transplantation, *Ex-vivo* lung perfusion, Brain-dead donor, Lung transplantation technique

## Abstract

**Background:**

Although lung transplantation is a well-accepted treatment for end-stage lung diseases patients, only 15%–20% of the brain-dead donors’ lungs are usable for transplantation. This results in high mortality of candidates on waiting lists. *Ex-vivo* lung perfusion (EVLP) is a novel method for better evaluation of a potential lung for transplantation.

**Objective:**

To report the first experience of EVLP in Iran.

**Methods:**

The study included a pig in Vienna Medical University, Vienna, Austria, and 4 humans in Masih Daneshvari Hospital, Tehran, Iran. All brain-dead donors from 2013 to 2015 in Tehran were evaluated for EVLP. Donors without signs of severe chest trauma or pneumonia, with poor oxygenation were included.

**Results:**

An increasing trend in difference between the pulmonary arterial pO_2_ and left atrial pO_2_, an increasing pattern in dynamic lung compliance, and a decreasing trend in the pulmonary vascular resistance, were observed.

**Conclusion:**

The initial experience of EVLP in Iran was successful in terms of important/critical parameters. The results emphasize on some important considerations such as precisely following standard lung harvesting and monitoring temperature and pressure. EVLP technique may not be a cost-effective option for low-income countries at first glance. However, because this is the only therapeutic treatment for end-stage lung disease, it is advisable to continue working on this method to find alternatives with lesser costs.

## INTRODUCTION

The first successful lung transplantation (LTx) was done in 1983 by the Toronto Lung Transplant group [1]. Approximately 4000 transplantations are performed annually worldwide, according to the International Society for Heart and Lung Transplantation registries [2].

Nowadays, lung transplantation is accepted as a well-established treatment for patients with end-stage lung disease. Although the number of lung transplant candidates has grown significantly, only 15%–20% of brain-dead donors’ lungs are usable for transplantation. This results in a high mortality among lung transplant candidates on waiting lists. Some European centers reported that up to 50% of lung transplant candidates die while on waiting lists [3, 4].

Greater susceptibility of the lungs to deleterious effects of brain death (*i.e.*, inflammatory response) and ICU complications (prolonged intubation, pneumonia, barotrauma and excessive crystalloid injection) results in low rate of available lungs as a transplant [5, 6]. However, in recent years, various strategies have been proposed to increase the number of transplanted lungs using marginal lung and non-heart-beating donor. There is however, evidence suggesting an increased incidence of some complications, such as primary graft dysfunction, in recipients of such marginal organs [7, 8]. 

To overcome two main limitations—scarcity of donor lungs and post-operative complications—a novel approach, namely *ex vivo* lung perfusion (EVLP), has been established by Steen, *et al* [9]. In this operation, poorly oxygenated lungs (p_a_O_2_ < 300 mm Hg with FIO_2 _100%) with pulmonary edema or atelectasis without established pneumonia, severe contusions or gross gastric aspiration are perfused *ex vivo* by a high oncotic pressure solution, the so-called “Steen solution,” and then ventilated for time points, and monitored on time. In condition with difference between the pulmonary arterial pO_2_ and the left atrial pO_2_ (defined as ΔpO_2_) is more than 350 mm Hg and other functional parameters (dynamic lung compliance, peak inspiratory pressure, pulmonary artery pressure, and pulmonary vascular resistance) are stable or improved after a minimum of 2 hours of EVLP, the lungs are considered as a candidate for transplantation [7]. 

For the first time using this method in 2005, a successful EVLP was performed on a 19-year-old brain-dead donor that was rejected for LTx by all lung transplant centers in the Lund University Hospital, Sweden. The recipient was a 70-year-old man with chronic obstetric pulmonary disease, who became socially very active after LTx. Unfortunately, he died of acute sepsis 11 months after the transplantation [9]. There have been several prospective completed and ongoing clinical trials of various EVLP techniques in Europe and North America. It can be addressed to HELP trial in Toronto, NOVEL and Perfusix trials in the USA, Vienna trial in Austria using Toronto method, INSPIRE and EXPAND trials in the USA and Europe with OCS method, and DEVELOP trial in the UK using Steen method [10]. 

Herein, we present our experience with this technique in Iran.

## MATERIALS AND METHODS

Study Design

This study included five cases: a pig in Vienna Medical University, Vienna, Austria, and four humans in Masih Daneshvari Hospital, Tehran, Iran. All brain-dead multi-organ donors from May 2013 to July 2015 in Tehran were evaluated for the inclusion criteria of lung transplantation or EVLP. The pig case was arranged to make the Iranian team qualified for EVLP. Theoretical and practical points including lung harvesting, antegrade and retrograde lung flushing, left-atrium (LA) and pulmonary artery (PA) anastomosis, cannulation, perfusion, ventilation, and periodic lung assessment were all discussed.

Inclusion and Exclusion Criteria

Donors younger than 65 years with history of smoking <20 pack/day, ratio of arterial pO_2_ (p_a_O_2_) to fraction of inspired oxygen (FIO_2_) <300 mm Hg, without any significant improvement by recruitment maneuver and chest radiographic findings suggestive of pulmonary edema, defined as bilateral interstitial infiltration, were included in this study. Exclusion criteria included donor lungs with established pneumonia, severe mechanical lung injury (*i.e.*, contusions in more than one lobe) or gross gastric aspiration.

*Ex Vivo *Lung Perfusion Technique

Toronto technique, used in this study, was developed by Cypel, *et al*, which allows prolonged perfusion, organ recovery and active treatment for organ repair along with lung assessment. More details of acellular EVLP technique are provided elsewhere [11]. 

Briefly, the circuit was primed with 2 liters of Steen solution (XVIVO, Vitrolife, Sweden). In addition, 500 mg of imipenem/cilastatin (Jaberebne Hayan Pharmaceutical Company, Tehran, Iran), 3000 IU of heparin (Caspian Tamin Pharmaceutical Company, Rasht, Iran), and 500 mg of methylprednisolone (Solu-Medrol, Sandoz Canada, Boucherville, Canada) were added to the perfusate solution. Table 1 demonstrates the Toronto initiation strategy.

**Table 1:** Strategy for initiation of *ex vivo* lung perfusion

Perfusion time (min)	0	10	20	30	40	50	60
Termination temp (°C)	20	30	32-35	37	37	37	37
Flow (% Calculated flow)	10	10	20	30	50	80	100
Ventilation	None	None	Start				Recruitment
Gas exchanger	None	None	Start				
Left arterial pressure (mm Hg)	3–5	3–5	3–5	3–5	3–5	3–5	3–5

After the first hour of EVLP, 500 mL of circulated perfusate was removed and replenished with 500 mL of fresh solution. Subsequently, 250 mL was exchanged every hour until the end of the procedure. After transferring lungs to the XVIVO chamber, the pulmonary artery and LA cannulas (XVIVO) were connected to the circuit and anterograde flow was started at 150 mL/min of the perfusate at room temperature. The temperature of the perfusate was then gradually increased to 37 °C. When the temperature of 32 °C was reached (usually >30 min), ventilation was started and the perfusate flow rate was increased gradually. A flow of mixed gas of N_2_, O_2_, and CO_2_ (Roham Gas, Iran) was used to deoxygenate and provide carbon dioxide to the inflow perfusate via a gas exchange membrane which was initiated at 1 L/min and adjusted according to the arterial blood gas analysis. In this method, mechanical ventilation is based on protective mode with perfusion flow limited to 40% of the cardiac output, pulmonary arterial pressure of 7–15 mm Hg, and a LA pressure of 5 mm Hg in a closed circuit. 

**Figure 1:**
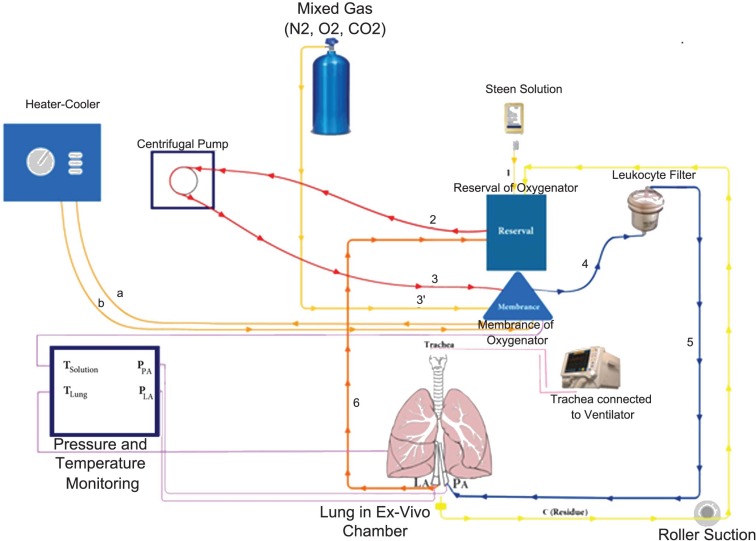
*Ex vivo* lung perfusion circuit. Circuit 1: Steen solution from bottle to reservoir of cardio-pulmonary bypass pump; Circuit 2: Steen circulated from reservoir to centrifugal pump; Circuit 3: circulation from centrifugal pump to membrane; Circuit 3': circulation of mixed gas from gas cylinder to membrane, circuit a: solution from membrane to heater-cooler, circuit b: solution from heater-cooler to membrane; Circuit 4: solution from membrane to leukocyte filter; Circuit 5: solution from leukocyte filter to pulmonary artery; Circuit 6: returning solution from left atrium to reservoir, circuit c: returning solution residue from chamber to reservoir through roller suction, purple circuit: solution and lung temperature monitoring

**Figure 2:**
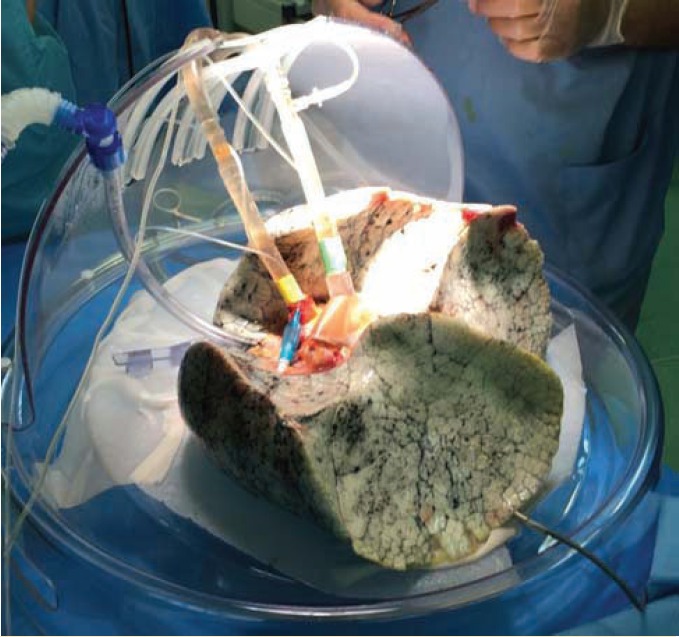
Lung on the EVLP system

At the final step, the lung block was cooled down in the circuit to 10 °C within a 10-min period. Thereafter, perfusion and ventilation were stopped (FIO_2_ increased to 50% for lung storage), and the trachea was clamped to maintain the lungs in an inflated state. The lungs were then statically preserved at 4 °C in Perfadex until transplantation. The maintenance strategy is presented in Table 2. Figures 1 and 2 show the circuit diagram and a lung on EVLP system, respectively.

**Table 2:** EVLP maintanace strategy settings

Measure	Setting
Tidal volume	6–8 mL/kg
PEEP	5 cm H_2_O
Respiratory rate	7 breaths/min
FIO_2_	21%
Flow rate	40% of estimated cardiac output
LAP	3–5 mm Hg
PAP	10–15 mm Hg
Recruitments	To PawP of 25 cm H_2_O

PEEP: positive end-expiratory pressure; FIO_2_: fraction inspired of oxygen; LAP: left atrial pressure; PAP: mean pulmonary artery pressure; Recruitments were performed by 2 inspiratory holds of 15 seconds to a PawP (peak airway pressure) of 25 cm H_2_O every hour.

*Ex Vivo* Functional Assessment

This method was done as described earlier [11]. The pH, pCO_2_, electrolytes, and glucose were maintained at physiological levels in the perfusate. For the functional assessment of *ex vivo*, the tidal volume was set at 10 mL/kg of donor body weight and 10 breaths/min for 5 min with an FIO_2_ of 1.0. Lung function was evaluated hourly during EVLP according to the following equations:

ΔpO_2_ = LA pO_2_ – PA pO_2_ (mm Hg),

and 

(in dynes·sec·cm^5^), dynamic compliance (in mL/cm H_2_O), and peak inspiratory pressure (in cm H_2_O). Radiography of the *ex vivo* lung and flexible bronchoscopy were performed at 1 and 3 hours of EVLP.

## RESULTS

A total of five cases including one pig and four humans were studied. An increasing trend in ΔpO_2 _was observed in all of the cases. In addition, there was an increasing trend in other functional parameters including dynamic lung compliance, and also a decreasing trend in the pulmonary vascular resistance. Although the observed improved trends were acceptable, they did not meet the end-point we had defined—a ΔpO2 = 350 mm Hg. In the pig, ΔpO_2_ increased up to 330 mm Hg; the lung compliance and pulmonary vascular resistance were acceptable. 

A 47-year-old brain-dead donor due to cerebrovascular accident was selected as the second case. All cannulations were connected to the circuit and normal saline was injected to the circulation instead of Steen. Soon, problems related to the anastomosis, cannulations, air leak, and liquid leak were observed. The third case was a 63-year-old non-smoker brain-dead due to a benign brain tumor who had been intubated for 9 days with a p_a_O_2_/ FIO_2_ of 125 mm Hg, pulmonary edema in both lungs and normal bronchoscopy findings. EVLP was started after 130 min of aortic clamp; the anastomosis was satisfying, but there were problems with air leak in the circuit and liquid leak from the site of lung biopsy that had been done for research purposes. At the end, foamy yellowish secretions, which were suggestive of pulmonary edema, were removed from the lungs. ΔpO_2_ increased from 19 to 110 mm Hg. PVR at first decreased from 780 to 360 but increased to 800 dynes·sec·cm^5^. Improved results were seen in the fourth case, a 43-year-old brain-dead donor due to hypertension crisis, with a p_a_O_2_/ FIO_2_ of 220 mm Hg that increased to 260 after recruitment maneuver, obvious atelectasis in right lung, and normal bronchoscopy findings. His lungs were been harvested in another center, kept in ice coalman for one hour, and finally underwent EVLP in Masih Daneshvari Hospital. Time between aortic clamp and starting EVLP was 180 min. Pressures were kept at an overall acceptable level in this case, however there were problems with temperature monitoring, especially during the first 30 min. ΔpO_2_ increased from 62 to 223 mm Hg, in addition, PVR decreased from 350 to 130 at the beginning and then increased to 444 dynes·sec·cm^5^. Lung dynamic compliance improved from 44 to 69 mL/cm H_2_O and stabilized around 45. 

In the fifth case, all principles of harvesting were accurately applied; prostacycline was carefully injected; sufficient amount of time was spared to let the lungs cool down during harvesting and before the onset of ELVP, however, a LA pressure of 3–5 mm Hg was not met and due to some technical problems a sudden shift of fluid into the lungs was occurred and so the process had to be terminated. Fortunately, the system was successfully set up, and promising results were growing by the hour. ∆pO_2_ in liquid samples was increasing so that it reached 230 mm Hg in the fifth hour, while it was 65 mm Hg at the onset. The details of perfusion evaluation of the third and fourth cases are shown in Tables 3 and 4, respectively.

**Table 3:** Perfusion evaluation of the third case of EVLP

Hours/min	30’	1h	1h30’	2h	2h30’	3h	3h30’	4h	4h30’	5h	5h30’	6h
Time	18:30	19	19:30	20	20:30	21	21:30	22	22:30	23	23:30	24
PA flow PAF (L/min)	0.5	0.9	1.2	1.4	1	0.5	0.8	0.9				
PA pressure PAP (mm Hg)	12	20	5	5	7	6	5	5	7	5	6	7
LA pressure LAP (mm Hg)	3	20	3	3	5	3	0	1	0	0	-1	0
PVR (dynes·sec·cm^5^)	782		600	360		800						
Mean Paw (cm H_2_O)	7	7		9		8		7		7		7
Peak Paw (cm H_2_O)	17	16		21		23		20		19		18
Plat. Paw (cm H_2_O)	12	13		14		14		10		10		11
Dyn. Compl. (mL/cm H_2_O)	49	50		45		41		49		55		68
Inflow (PA) pO_2_ (mm Hg)		167		170		169		168		109		118
Inflow (PA) pCO_2_ (mm Hg)		30		23		16		18		23		26
Inflow (PA) pH		7.14		7.14		7.16		7.10		6.95		6.88
Outflow (PV) pO_2 _(mm Hg)		186		176		219		240		219		298
Outflow (PV) pCO_2_ (mm Hg)		33		22		18		18		13		18
Outflow (PV) pH		7.11		7.14		7.14		7.10		7.06		6.94
pO_2_ difference (mm Hg)		19		6		50		72		110		
FIO_2_(%) (21%/100%)	21%	50%	50%	100%	100%	100%	50%	50%	50%	50%	50%	50%
Steen backfilling		500	250	250	250	250	250	250	250	250	250	

**Table 4:** Perfusion evaluation of the forth case of EVLP

Hours/min	30’	1h	1h30’	2h	2h30’	3h	3h30’	4h	4h30’	5h	5h30’	6h
Time	19:00	19:30	20:00	20:30	21:00	21:30	22:00	22:30	23:00	23:30	24:00	24:30
PA flow PAF (L/min)	1	6	8	9	10							
PA pressure PAP (mm Hg)	5	6	11		-3			12		13		
LA pressure LAP (mm Hg)	1	3	8					-4		3		
PVR (dynes·sec·cm^5^)	355	133	133					711		444		
Mean Paw (cm H_2_O)	6	8	8	7		11		15		13		7
Peak Paw (cm H_2_O)	12	15	17	15	20			29		25		
Plat. Paw (cm H_2_O)	11	12	15	13	17			25		21		
Dyn. Compl. (mL/cm H_2_O)	44	69	57	58	52	48	50	43		45		
Inflow (PA) pO2 (mm Hg)	85	122		303				319		326		
Inflow (PA) pCO_2_ (mm Hg)	28	19		13				14		10		
Inflow (PA) pH	7.07	7.13		7.13				7.04		7.02		
Outflow (PV) pO2 (mm Hg)	147	209		110				90		93		
Outflow (PV) pCO_2_ (mm HG)	22	16		20				25		21		
Outflow (PV) pH	7.14	7.20		7.06				6.94		6.90		
pO_2_ difference (mm Hg)	62	87		197				205		233		
FIO_2_ (%) (21%/100%)	21%	50%	50%	100%	100%	100%	50%	50%	50%	50%	50%	50%
Steen backfilling		500	250	250	250	250	250	250	250	250	250	
PEEP	5	5	5	5	5	5	5	5	5	10	5	5
I/E	1/2	1/2	1/2	1/2	1/2	1/2	1/2	1/2	1/2	1/2	1/2	½
Tidal volume	450	500	550	550	550	550	550	600	650	700		
Stat. Compl. (mL/cm H_2_O)	53	97	70	79	64			55				

## DISCUSSION

Shortage of suitable lung and post-operative complications such as primary graft dysfunction motivated our team to establish a study for approaching the *ex vivo* normothermic perfusion. 

We have reported herein strategy for EVLP of the donor lung. Some technical hints have been considered as key elements to reach a successful EVLP. EVLP is not a single technique; it is a collection of many arts and methods that should be carefully followed to improve a marginal donor lung. In the current study, the animal model provided information to start the human model. In the animal model, we reached a successful trend in ΔpO_2_ status and the functional parameters. 

Some successful lung transplantation teams have had an EVLP success rate of about 60% [7]. Therefore, it can be concluded that our initial experience indicated a considerable improvement in the initial establishment of this technique in Iran. To conduct an EVLP system in a center, it is mandatory for the team members to be highly qualified. Particularly, for low experienced centers, it is advisable to consider selection of donor and avoid choosing extreme marginal lungs in the initial steps. The team should follow the standard lung harvesting such as a conventional lung transplantation including core and surface cooling and administration of prostaglandin during the harvest. Another important point is to pay attention to storing the lung at 4 °C between harvest and EVLP to decrease cellular metabolism at the basal level, and also not to start EVLP immediately after lung removal. It is necessary to monitor the lung and the solution temperature and pressure precisely. A comprehensive discussion about the whole process of EVLP in terms of the four phases are available elsewhere [12].

Based on the initial results, EVLP can lead to a great change and a remarkable progression in lung transplantation in Iran, given the well-experienced team. It is suggested that this operation to be continued seriously to achieve more significant successful results in the near future. As lung transplant is not currently under coverage of insurance companies in Iran, the average cost of a conventional lung transplantation and an EVLP is estimated at US$ 22,800 and US$ 15,000, respectively. 

Saving human’s life is the reason why such services should be supported. Lung transplantation, like heart and liver transplantation, is a life-saving process. In 2014, only 14 lung transplantations were done in Iran. About 480 patients with end-stage lung disease are referred to lung transplant clinics annually, of whom 160 can be listed as active candidates. The first and the most important obstacle encountered in this way is the shortage of suitable organs. Although 260 brain-dead patients were reported last year in Tehran, only 5%–10% of them had suitable lungs for transplantation. Lungs were rejected mostly because of insufficient ICU care, and also during trauma as the most common cause of brain death in Iran; the rate of aspiration pneumonia is also high.

One of the limitations of our study was the low sample size of this study that was attributed to the financial issues. It is highly recommended to conduct future studies on more cases in order to gain a better understanding as well as reconditioning brain-dead lungs safely for lung transplantation and immunological investigations.
